# H-Ras Nanocluster Stability Regulates the Magnitude of MAPK Signal Output

**DOI:** 10.1371/journal.pone.0011991

**Published:** 2010-08-05

**Authors:** Barak Rotblat, Liron Belanis, Hong Liang, Roni Haklai, Galit Elad-Zefadia, John F. Hancock, Yoel Kloog, Sarah J. Plowman

**Affiliations:** 1 Department of Neurobiology, George S. Wise Faculty of Life Sciences, Tel Aviv University, Tel Aviv, Israel; 2 Department of Integrative Biology and Pharmacology, University of Texas Health Science Center-Houston, Houston, Texas, United States of America; University of Illinois at Chicago, United States of America

## Abstract

H-Ras is a binary switch that is activated by multiple co-factors and triggers several key cellular pathways one of which is MAPK. The specificity and magnitude of downstream activation is achieved by the spatio-temporal organization of the active H-Ras in the plasma membrane. Upon activation, the GTP bound H-Ras binds to Galectin-1 (Gal-1) and becomes transiently immobilized in short-lived nanoclusters on the plasma membrane from which the signal is propagated to Raf. In the current study we show that stabilizing the H-Ras-Gal-1 interaction, using bimolecular fluorescence complementation (BiFC), leads to prolonged immobilization of H-Ras.GTP in the plasma membrane which was measured by fluorescence recovery after photobleaching (FRAP), and increased signal out-put to the MAPK module. EM measurements of Raf recruitment to the H-Ras.GTP nanoclusters demonstrated that the enhanced signaling observed in the BiFC stabilized H-Ras.GTP nanocluster was attributed to increased H-Ras immobilization rather than to an increase in Raf recruitment. Taken together these data demonstrate that the magnitude of the signal output from a GTP-bound H-Ras nanocluster is proportional to its stability.

## Introduction

Ras GTPases are membrane-associated proteins that regulate signaling pathways controlling cell growth and differentiation. Ras proteins diffuse rapidly on the inner leaflet of the plasma membrane [1], [2] where they organize into nanoclusters that are critical for Ras signal transduction [3], [4], [5]. All Ras isoforms are also mobilized to and from the cell membrane and can relay signals from intracellular membrane compartments including the Golgi complex, endoplasmic reticulum (ER), mitochondria and endosomes [6], [7], [8], [9], [10]. The contribution of cellular membranes to the fidelity of Ras signaling has been best characterized in the physiological context of epidermal growth factor (EGF) receptor stimulation of the Ras-Raf-MEK-ERK signaling cascade at the cell membrane.

In the absence of growth factor receptor activation, H-Ras is GDP bound and organized into cholesterol-dependent nanoclusters that have radii of ∼12 nm [3], [4]. Following growth factor stimulation H-Ras becomes GTP loaded and undergoes a lateral shift into cholesterol-independent nanoclusters that have radii of 6–8 nm [3], [4]. Ras nanoclusters are essential for high fidelity signal transduction across the plasma membrane because these nanoclusters act as signaling platforms to which cytosolic effectors such as Raf are recruited [5]. Approximately 60% of Ras.GTP proteins are randomly distributed on the inner leaflet of the plasma membrane while the remaining 40% are organized into cholesterol-independent nanoclusters [4]. The structural elements within H-Ras that are required to regulate GTP lateral segregation have been well characterized and include: farnesylation, dual palmitoylation, amino acid sequences in region 1 of the hypervariable region (HVR) (residues 166–172), correct spacing of region 1 from the membrane anchor provided by region 2 (residues 173–179) of the HVR and basic residues in helix α4 [9], [11], [12], [13]. Single fluorophore video tracking in live cells shows that Ras signaling platforms are short-lived (<1 s)[14].

We identified galectin-1 (Gal-1) as a critical scaffold for the formation of H-Ras.GTP nanoclusters [15], [16], [17]. Following H-Ras GTP-loading Gal-1 is recruited from the cytosol to the plasma membrane where it forms a complex with H-Ras GTP-bound molecules. It is these complexes that form the basic building block for H-Ras.GTP nanoclusters. Over-expression of Gal-1 increases the level of H-Ras.GTP nanoclustering, providing more Raf recruitment sites in response to EGF and enhancing ERK activation [15], [17]. The time course of Gal-1 and H-Ras interaction [17] is similar to that of EGF-stimulated H-Ras GTP-loading [15], [18]. Together these observations point to the physiological relevance of Gal-1-driven H-Ras.GTP nanoclustering. Gal-1 also acts as a molecular chaperone that contributes to H-Ras trafficking by returning depalmitoylated H-Ras to the Golgi complex for repalmitoylation [17]. Thus Gal-1 appears to have a dual role in H-Ras signal transduction by first regulating H-Ras.GTP nanocluster formation and second by serving as a chaperone for H-Ras trafficking to the Golgi complex [17].

Based on these earlier studies we propose that H-Ras signal-output depends on the lifetime of Gal-1-driven H-Ras.GTP nanoclusters. Therefore we hypothesize that the strength of H-Ras.GTP and Gal-1 binding will determine the amplitude and duration of the signal output by regulating the duration of H-Ras nanocluster formation. In the current manuscript, we test our hypothesis using fluorescence recovery after photobleaching (FRAP) to determine the dynamics of H-RasG12V-Gal-1 complexes on the plasma membrane of live cells, and immunogold EM spatial mapping to determine recruitment of Raf to H-RasG12V-Gal-1 nanoclusters. We demonstrate that artificially stabilizing Gal-1-H-Ras.GTP nanoclusters on the plasma membrane generates a signal output that is three times greater than that of transiently immobilized nanoclusters. Thus our results establish for the first time that the lifetime of the Ras nanocluster is the critical determinant of signal output from the nanocluster.

## Results

### Gal-1 induces transient immobilization of GFP-H-RasG12V

**Figure 1:**
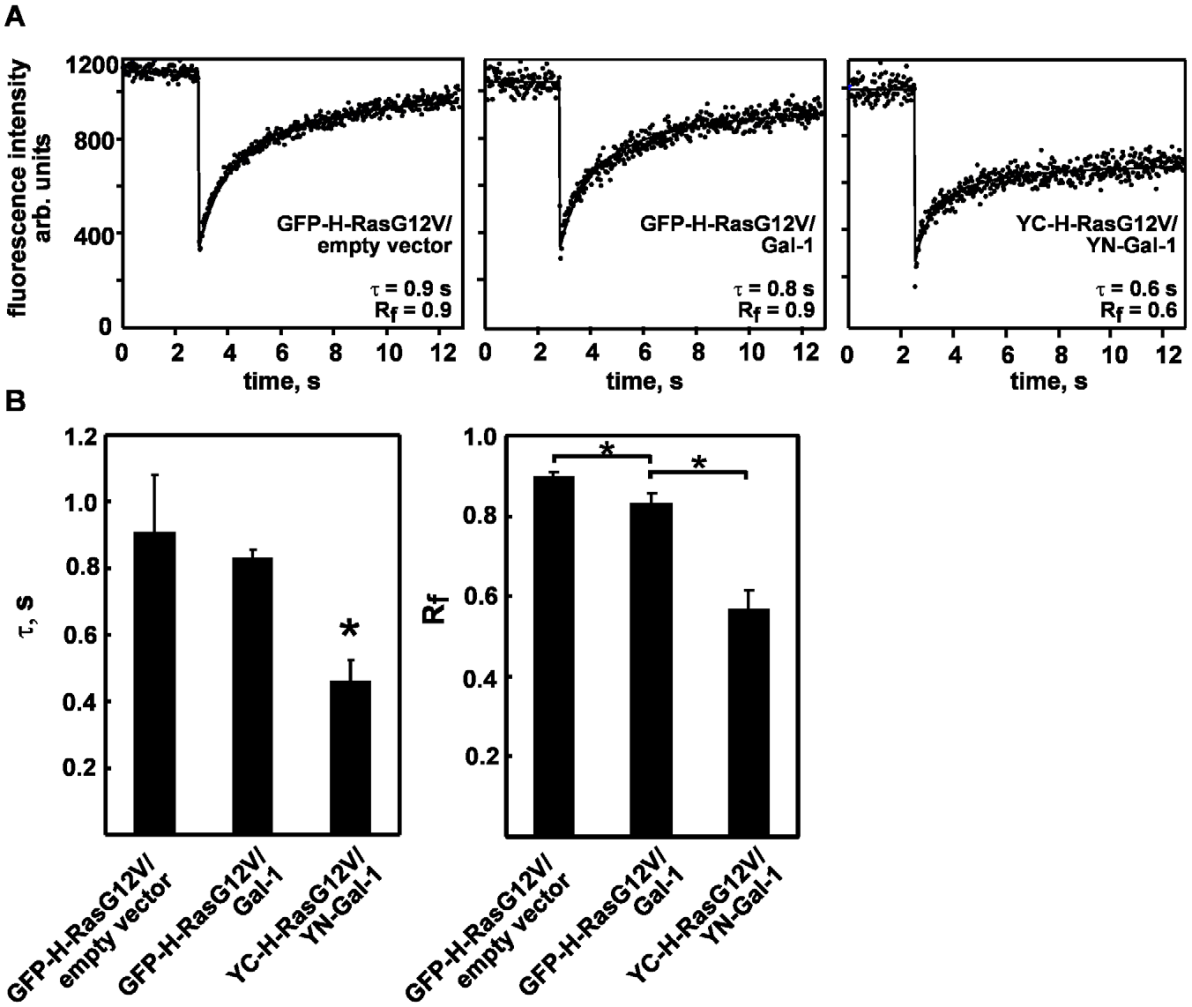
GFP-H-RasG12V and Gal-1 form mobile and immobile complexes on the plasma membrane. BHK cells transiently co-expressing GFP-H-RasG12V/empty control, GFP-H-RasG12V/Gal-1, GFP-H-Ras/empty vector, GFP-H-Ras/Gal-1, YC-H-RasG12V/YN-Gal-1 or YC-H-Ras/YN-Gal-1 were analyzed by FRAP. A. Typical FRAP curves recorded with a 40× objective in the indicated co-transfectants are shown. The half time of fluorescence recovery (µ) and mobile fraction (Rf) of these typical curves are indicated. B. Statistical analysis of µ and Rf recorded with 40× objective in each of the cotransfectants. Bars represent the mean values (± SEM, n = 15–60). * *P*<0.001.

Ras proteins undergo periods of transient immobility on the plasma membrane [14], which we propose correspond to Ras nanoclusters. Gal-1 regulates the formation of H-Ras.GTP nanoclusters [17]. Therefore we investigated how over- expression of Gal-1 might affect the mobility of H-Ras.GTP on the plasma membrane. Using FRAP measurements, we showed that GFP-H-RasG12V exhibited very rapid motility on the plasma membrane, characterized by half time of fluorescence recovery (τ) of 0.9 sec (40× objective) and a high mobile fraction Rf>0.93 (Figure 1A and B). These measurements are in agreement with previous reports [9], [11], [19]. In the presence of Gal-1, the τ values recorded for GFP-H-RasG12V were not significantly different from those recorded for GFP-H-RasG12V in the absence of Gal-1 (*P* = 0.5, Figure 1A and B). However, Gal-1 over-expression caused a significant (P = 0.003) decrease in the GFP-H-RasG12V mobile fraction (Rf = 0.82±0.02) compared with control cells expressing GFP-H-RasG12V alone (Figure 1A and B). Thus expression of Gal-1 causes a small but significant fraction (18%) of GFP-H-RasG12V to become immobile.

**Figure 2:**
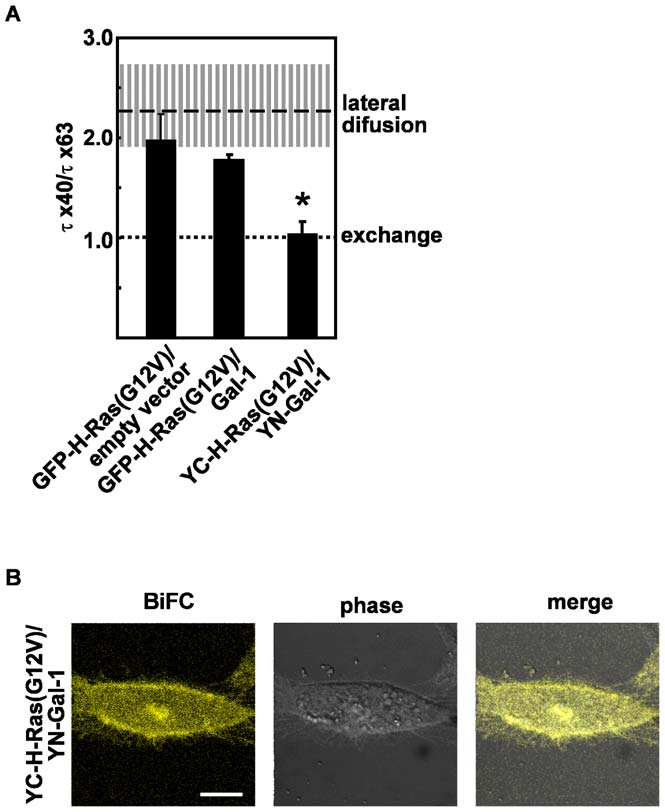
FRAP beam-size analysis of GFP-H-RasG12V- Gal-1 complexes. A. The various co-transfectants described in Figure 1 were subjected to FRAP measurements that were performed using two beam sizes generated by 63× and 40× objectives. Bars represent the mean (± SEM) of τ ratios (τ (40×)/τ (63×)) derived from 15–60 measurements for each of the co-transfectants. The experimentally determined ratio between the areas illuminated by the laser beam using the two objectives (n = 23) is indicated by the striped horizontal line (mean). The SEM is denoted by the vertical lines. * Statistical significance compared with empty vector control, *P*<0.001. B. YC-H-RasG12V interacts with YN-Gal-1 in BHK cells. BHK cells were transfected with the indicated BiFC vectors and were examined by confocal microscopy.

To examine whether the mobile fraction of GFP-H-RasG12V-Gal-1 complexes were firmly associated with the plasma membrane, we used the FRAP beam-size test to distinguish between lateral diffusion, indicative of relatively stable membrane association, and exchange of proteins between the plasma membrane and the cytoplasm [9], [11], [20]. The FRAP curves were generated by using a 40× objective (Gaussian radius of 1.36 µm) and a 63× objective (Gaussian radius of 0.85 µm) [9], [11], [20]. In line with previous results [9], [11], GFP-H-RasG12V exhibited pure lateral diffusion on the plasma membrane, evident by a τ (40×)/τ (63×) ratio of 1.9±0.2, not significantly different from the ratio between the areas illuminated by the two objectives (2.3±0.4, *P*>0.1; Figure 2A). GFP-H-RasG12V also exhibited pure lateral diffusion on the plasma in the presence of Gal-1 (Figure 2A); the recorded τ (40×)/τ (63×) ratio was 1.7±0.2, not significantly different from the ratio recorded in the control GFP-H-RasG12V cells (*P* = 0.07). The calculated diffusion coefficients of GFP-H-RasG12V (*D* = 5.15×10^−9^) and of GFP-H-RasG12V-Gal-1 complexes (*D* = 5.12×10^−9^) are almost identical, consistent with weak dependence of molecular diameter on diffusion rate. Thus all GFP-H-RasG12V-Gal-1 complexes exhibit relatively stable interactions with the plasma membrane of live cells.

### The lifetime of immobile H-Ras.GTP nanoclusters depend on the strength of Gal-1 interaction with H-RasG12V

Next we examined whether immobilization of H-Ras.GTP-Gal-1 complexes on the plasma membrane was dependent upon the strength of H-Ras.GTP and Gal-1 interactions. To test this possibility we used the YN-Gal-1 and YC-H-RasG12V constructs reported previously [17]. In our previous report we carefully characterized the specificity of the bimolecular fluorescence complementation (BiFC). Our data demonstrated that YN-Gal-1 interacts with GTP- but not GDP-loaded H-Ras demonstrating that BiFC does not perturb the mechanisms regulating H-Ras Gal-1 interaction [17]. Due to strong interaction between the two YFP fragments YN-Gal-1 and YC-H-RasG12V formed longed-lived highly stable complexes upon interaction. YN-Gal-1 and YC-H-RasG12V yielded strong BiFC upon binding and were localized properly to the plasma membrane and the Golgi complex as reported previously (Figure 2B) [17].

FRAP experiments demonstrated that YC-H-RasG12V-YN-Gal-1 complexes had only a relatively small mobile fraction (Rf = 0.56±0.05) (Figures 1A and B) which was significantly smaller (*P*<0.01) than that recorded for GFP-H-RasG12V in the GFP-H-RasG12V/Gal-1 co-transfectants (Rf = 0.82±0.02). Thus, the fraction of immobile YC-H-RasG12V-YN-Gal-1 complexes on the plasma membrane (0.4±0.05) was significantly higher than that of GFP-H-RasG12V (0.18±0.02) recorded on the plasma membrane of GFP-H-RasG12V/Gal-1 co-transfectants. Together these data suggest that the strength of the interaction between H-RasG12V and Gal-1 determines the mobility of H-RasG12V on the plasma membrane.

The mobile fraction of the YC-H-RasG12ṼYN-Gal-1 complexes had a relatively short recovery time (τ value of 0.45±0.06 s), significantly shorter than that of GFP-H-RasG12V in GFP-H-RasG12V/Gal-1 co-transfectants (Figure 1 A and B, *P*<0.01). FRAP beam-size analysis demonstrated that this short recovery half-time was due to weaker association with the plasma membrane and exchange of YC-H-RasG12ṼYN-Gal-1 complexes between the cytosol and the membrane (τ (40×)/τ (63×) ratio of 1.0) (Figure 2A).

Taken together these data show that the highly stable YC-H-RasG12V-YN-Gal-1 complexes are comprised of two populations: one that is immobile on plasma membrane and the other that is highly mobile and exhibits weak interactions with the plasma membrane.

### Immobilization of H-RasG12V-Gal-1 complexes increases signal output

**Figure 3:**
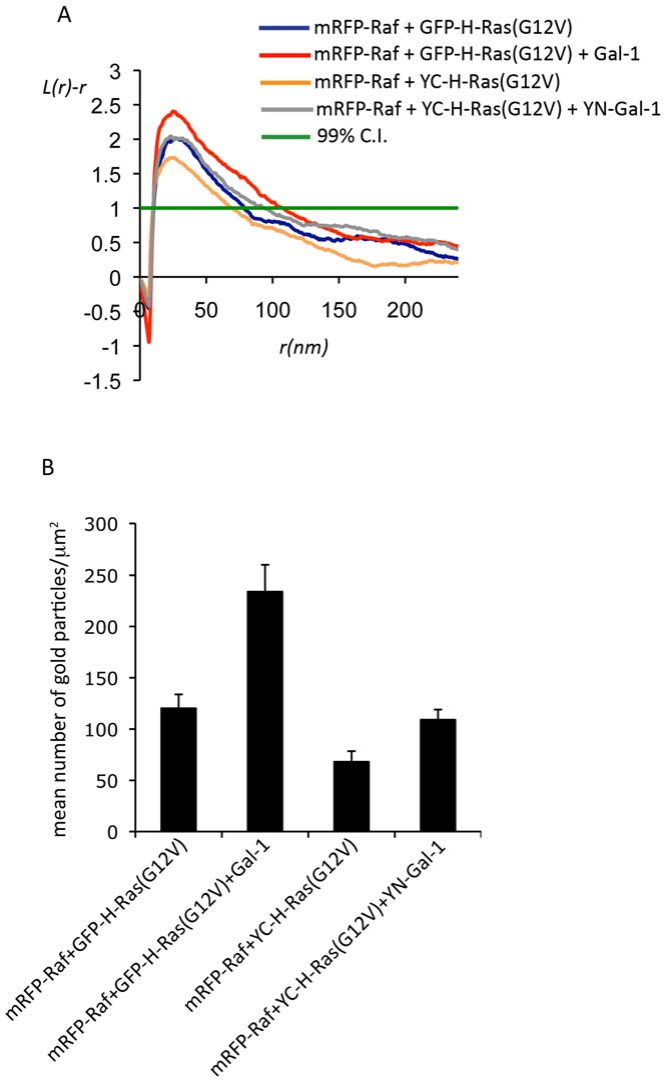
Expression of Gal-1 increases Raf recruitment to the inner leaflet of the plasma membrane by H-RasG12V. Plasma membrane sheets were generated from serum-starved cells expressing the following combinations of constructs: GFP-H-RasG12V/mRFP-Raf, GFP-H-RasG12V/mRFP-Raf/Gal-1, YC-H-RasG12V/mRFP-Raf and YC-H-RasG12V/YN-Gal-1/mRFP-Raf. The plasma membrane sheets were labeled with anti-mRFP antibody conjugated to 5 nm gold to detect mRFP-Raf plasma membrane recruitment. A. The spatial distribution of the gold labelling was analyzed using Ripley's K-function. Maximum L(r)-r values above the 99% confidence interval (CI) for complete spatial randomness (CSR) indicate clustering at the value of r (supr). Univarate K-functions are weighted means (n>8) standardized on the 99% CI. B. Analysis of the number of mRFP-Raf proteins recruited to the plasma membrane. Bars represent the mean number of gold particles/µm^2^ (± SEM).

H-Ras.GTP nanoclusters are the sites of effector recruitment [17] and H-Ras.GTP nanocluster immobilization has been proposed to enhance Ras signaling. Therefore we hypothesized that the BiFC-induced immobilization of YC-H-RasG12V-YN-Gal-1 complexes would result in enhanced H-Ras signal output. To address this hypothesis we first examined whether YC-H-RasG12V-YN-Gal-1 nanoclusters recruited mRFP-Raf to the plasma membrane like H-RasG12V-Gal-1 nanoclusters [17]. Indeed co-expression of GFP-H-RasG12V with Gal-1 or YC-H-RasG12V with YN-Gal-1 increased mRFP-Raf membrane recruitment and nanoclustering (Figure 3 and S1). Expression of Gal-1 in the presence of GFP-H-RasG12V increased mRFP-Raf immunogold plasma membrane labeling 1.9 fold compared to cells expressing GFP-H-RasG12V alone. We calculated a similar fold change in mRFP-Raf immunogold labeling in cells co-expressing YC-H-RasG12V and YN-Gal-1 compared to YC-H-RasG12V alone (1.6 fold, Figure 3).

**Figure 4:**
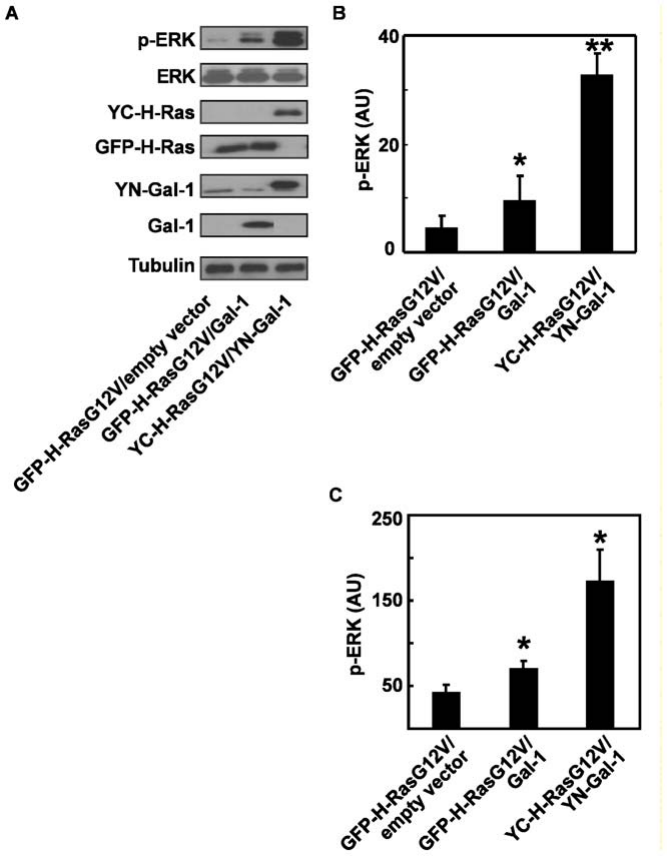
Enhanced activation of ERK by YC-H-RasG12ṼYN-Gal-1 complexes. HEK 293 or BHK cells were co-transfected with GFP-H-RasG12V/empty vector, GFP-H-RasG12V/Gal-1 or YC-H-RasG12V/YN-Gal-1 and lysed 48 h later. Aliquots of the cell lysates were subjected to SDS-PAGE followed by immunoblotting with anti-phospho-ERK, anti-ERK, anti-pan-Ras, anti-Gal-1 and anti-tubulin (loading control) antibodies. Typical immunoblots representing one of four experiments performed with HEK 293 cell are shown in panel A and quantitative densitometry of the levels of phospho-ERK (arbitrary units, means ± SEM, n = 4) are shown in panel B. *P<0.05, GFP-H-RasG12V/Gal-1 vs. GFP-H-RasG12V/empty vector; **P<0.0001, YC-H-RasG12V/YN-Gal-1 vs. GFP-H-RasG12V/Gal-1. Results obtained with BHK cells (arbitrary units, mean ± SEM, n = 2, *P<0.05) are shown in panel C.

Next we examined how the immobilization of YC-H-RasG12V-YN-Gal-1 complexes on the plasma membrane affected signal transduction. Co-expression of YC-H-RasG12V and YN-Gal-1 lead to a significant increase in pERK production compared to cells co-expressing YFP-H-RasG12V and Gal-1. This was the case when HEK 293 (*P*<0.0001, Figure 4A and B) or BHK cells were employed (Figure 4C). Similar results were observed in cells expressing YN-Gal-1 and GFP-H-RasG12V or YC-H-RasG12V (Figure S2). Thus even though stable YC-H-RasG12V-YN-Gal-1 complexes do not appear to recruit more Raf, the stability of the complex enhances the magnitude of signal output.

## Discussion

H-Ras.GTP nanoclusters are the sites to which cytosolic effectors such as Raf are recruited and activated to relay robust signals via the Raf-MEK-ERK pathway [3], [4], [5], [21]. We have shown that Gal-1 drives the formation and is an integral component of H-Ras.GTP nanoclusters [17]. Specifically EGF stimulates the formation of transient H-Ras.GTP-Gal-1 nanoclusters and the reversible recruitment of Raf. Here we test whether the magnitude of H-Ras signal-output is dependent upon the dynamics of H-Ras.GTP nanocluster formation, which we propose are driven by the lifetime of Gal-1 interactions with H-Ras.GTP.

In the current manuscript, we combined FRAP and immunogold EM spatial mapping to determine how the dynamics of H-RasG12V-Gal-1 complex formation on the plasma membrane effects Raf recruitment and signal output under two extreme conditions. In the first condition, interaction between GFP-H-RasG12V and Gal-1 is transient and nanocluster lifetime is short. In the second condition, the interaction between YC-H-RasG12V and YN-Gal-1 is stable and therefore the lifetime of the nanocluster is likely to be much longer. We show that Gal-1 expression leads to the formation of a small but significant fraction of immobile H-RasG12V/Gal-1 nanoclusters (18%). In contrast the formation of BiFC between YN-Gal-1 and YC-H-RasG12V markedly increases the immobile fraction to ∼40%. Importantly the YN-Gal-1 and YC-H-RasG12V complexes are immobile for >12 secs. The fraction of YC-H-RasG12V/YN-Gal-1 complexes that are immobile (∼40%) matches precisely the fraction of H-Ras.GTP nanoclusters on the inner plasma membrane as determined previously by the snap-shot EM-spatial mapping [4]. Murakoshi et al. [14], using single-fluorophore tracking microscopy reported that ∼30% H-RasG12V molecules on the inner plasma membrane exhibit an immobile period, and it is to these immobile Ras molecules that downstream effectors are recruited. The fraction of immobile Ras proteins reported by Murakoshi is close to the proportion of the immobile YN-Gal-1-YC-H-RasG12V complexes (40%) reported here. Therefore by combining these data we propose that the immobile YN-Gal-1-YC-H-RasG12V complexes detected in the current manuscript in fact represent active H-Ras.GTP nanoclusters.

The highly stable YN-Gal-1-YC-H-RasG12V complexes recruit Raf to the inner leaflet of the plasma membrane and generate 3 times more ppERK production than the short-lived Gal-1-GFP-H-RasG12V nanoclusters. Immuno-EM demonstrates that the respective H-RasG12V nanoclusters recruit the same proportion of Raf raising the question of how do YC-H-RasG12V/YN-Gal-1 nanoclusters generate increased signal output. At this point it is important to re-iterate that the immobile YC-H-RasG12V-YN-Gal-1 complexes exhibit zero mobility for a long period of time (>12 s, Figure 1 A). Therefore although each type of nanocluster is only able to recruit the same proportion of Raf at any one time because there are a finite number of Raf interaction sites, the YC-H-RasG12V/YN-Gal-1 nanoclusters exhibit enhanced lifetime and therefore we propose are able to recruit a larger number of Raf molecules over the lifetime of the cluster compared to GFP-H-RasG12V-Gal-1 nanoclusters. Thus our results establish that the strength of interaction between Gal-1 and H-Ras determines the lifetime of Gal-1-H-Ras.GTP nanoclusters and thus the magnitude of signal output.

In addition to the immobile YC-H-RasG12V-YN-Gal-1 nanoclusters a second, highly mobile fraction, of stable YC-H-RasG12V-YN-Gal-1 complexes was recorded on the plasma membrane. We propose that this highly mobile fraction represent complexes between YC-RasG12V and YN-Gal-1 that have not been incorporated into nanoclusters. The mobile YC-H-RasG12V-YN-Gal-1 complexes undergo rapid exchange between the plasma membrane and the cytosol. H-Ras undergoes a palmityolation-depalmitoylation cycle that promotes trafficking from the plasma membrane to the Golgi complex [7], [8], [9]. Our data suggest that the organization of H-Ras.GTP into nanoclusters protects the palmitate groups from the action of thioesterases thus stabilizing the interaction of H-Ras with the plasma membrane.

## Methods

### Plasmids

All GFP, YN and YN fusion constructs were prepared as detailed previously [17].

### Cell culture and imaging

Baby Hamster Kidney (BHK) cells were grown and/or transfected as detailed earlier [22]. Briefly, BHK cells were plated in 6 well plates (respectively 1.5×105 and 1×105 cell per well). The cells were transfected with a total of 2 µg DNA using 4 µl of jetPEI reagent according to the manufacturer's instructions. For live cells imaging BHK cells were plated on cover slips and transfected as described 24 hours later. Forty-eight hours after transfection the cells were used for live cell imaging. The medium was then supplemented with 10 mM HEPES pH 7.2 and the plates were kept for 20 min at room temperature. The cover slips were then placed on a camera microslide (Superior, Germany) filled with Hank's balanced salt solution supplemented with 20 mM HEPES, pH 7.2. Live cells were analyzed by Zeiss LSM 510 confocal microscope fitted with yellow fluorescence filter for detection of BiFC or were used for FRAP as detailed below.

### Immunoblotting

Whole cell lysates were generated from HEK293T cells (8×105 cells) co-transfected with the indicated constructs. Cell lysates were subjected to Western immunoblotting with specific antibodies to phospho-ERK (Santa Cruz Biotechnology, Inc., Santa Cruz, CA), ERK (Santa Cruz Biotechnology, Inc., Santa Cruz, CA), Ras (pan-ras, Calbiochem, La Jolla, CA) and Gal-1 as detailed earlier [15]. The bands were quantified by densitometry using TINA software as described previously [15].

### Fluorescence recovery after photobleaching (FRAP)

BHK cells were plated on glass coverslips placed in 35-mm dishes at a density of 1.5×105 cells per dish. After incubation for 24 h, the cells were transfected with each of the vectors encoding the various GFP- or YN tagged H-Ras proteins and Gal-1 or YC-Gal-1 proteins. Plasma membrane Spot-FRAP studies were conducted 48 h post-transfection as described earlier [9], [11]. All experiments were conducted at 22°C, in Hank's balanced salt solution supplemented with 20 mM HEPES, pH 7.2. The monitoring Argon ion laser beam (488 nm, 1.2 microwatt) was focused through the microscope (Zeiss Universal) to a Gaussian radius of 0.85±0.02 mm (63× objective) or 1.36±0.04 mm (40× objective). A brief pulse (6 milliwatts, 4–6 ms for the 63× objective, and 10–20 ms for the 40× objective) bleached 50–70% of the fluorescence in the illuminated region. The time course of fluorescence recovery was followed by the attenuated monitoring beam. The apparent characteristic fluorescence recovery time (τ) and the mobile fraction (Rf) were derived by nonlinear regression analysis, fitting to a lateral diffusion process with a single ˙τ value [23].

### Electron Microscopy

Apical plasma membrane sheets were prepared, fixed with 4% PFA, 0.1% glutaraldehyde and labeled with affinity purified anti-mRFP antibody coupled directly to 5 nm gold as described previously [3], [4]. Digital images of the immunogold labeled plasma membrane sheets were taken at 100,000× magnification in an electron microscope (Jeol 1011). Intact 1 µm^2^ areas of the plasma membrane sheet were identified using Image J and the (x,y) coordinates of the gold particles determined as described [3], [4]. Ripley's K-function was calculated using the (x,y) coordinates and then standardized on the 99% confidence interval (CI) for a random pattern as described [3], [4]. Bootstrap tests to examine differences between replicated point patterns were constructed exactly as described [24] and statistical significance evaluated against 1000 bootstrap samples.

## Supporting Information

Figure S1 Electron micrographs showing plasma membrane sheets labeled with anti-mRFP gold. Representative plasma membrane sheet generated from BHK cells expressing A) GFP-H-RasG12V, Gal-1 and mRFP-Raf-1 or B) YC-H-RasG12V, YN-Gal-1 and mRFP-Raf-1 labeled with anti-mRFP primary conjugated directly to 5 nm gold. The x,y co-ordinates of the gold pattern were used to calculate Ripley's k-function (see figure 3A). Bars represent 200 nm and 500 nm respectively.
(2.34 MB TIF)

Figure S2 HEK293 cells were co-transfected with YN-Gal1 and GFP-H-RasG12V or YC-H-RasG12V and lysed 48 h later. Aliquots of the cell lysets were subjected to SDS-PAGE followed by immunoblotting with anti-phospho-ERK, anti-ERK, anti-pan-Ras and anti-Gal1 antibodies. Typical immunoblots performed using HEK293 cell are shown (rite) and quantitative densitometry of the levels of phosphor-ERK vs. Ras levels (arbitrary units, means ± SEM, n = 4) are shown (left). P<0.05.
(0.20 MB TIF)
