# Supplementary figures and images for: H-Ras Nanocluster Stability Regulates the Magnitude of MAPK Signal Output
Source: PLoS One. 2010 Aug 5;5(8):e11991. doi: 10.1371/journal.pone.0011991 (PMC2916832; doi:10.1371/journal.pone.0011991)

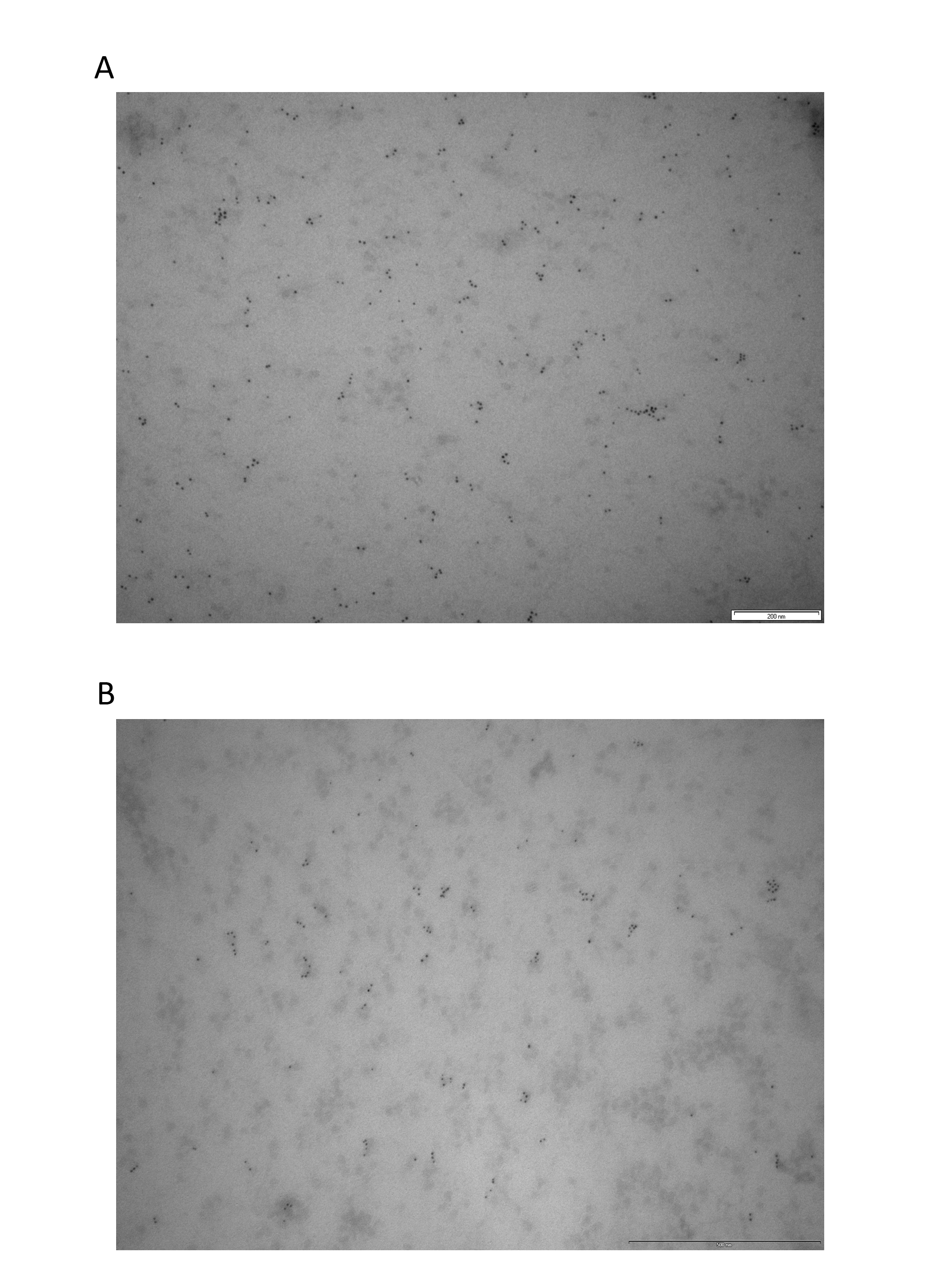

Supplement: Figure S1 — Electron micrographs showing plasma membrane sheets labeled with anti-mRFP gold. Representative plasma membrane sheet generated from BHK cells expressing A) GFP-H-RasG12V, Gal-1 and mRFP-Raf-1 or B) YC-H-RasG12V, YN-Gal-1 and mRFP-Raf-1 labeled with anti-mRFP primary conjugated directly to 5 nm gold. The x,y co-ordinates of the gold pattern were used to calculate Ripley's k-function (see figure 3A). Bars represent 200 nm and 500 nm respectively. (2.34 MB TIF) [file pone.0011991.s001.tif]

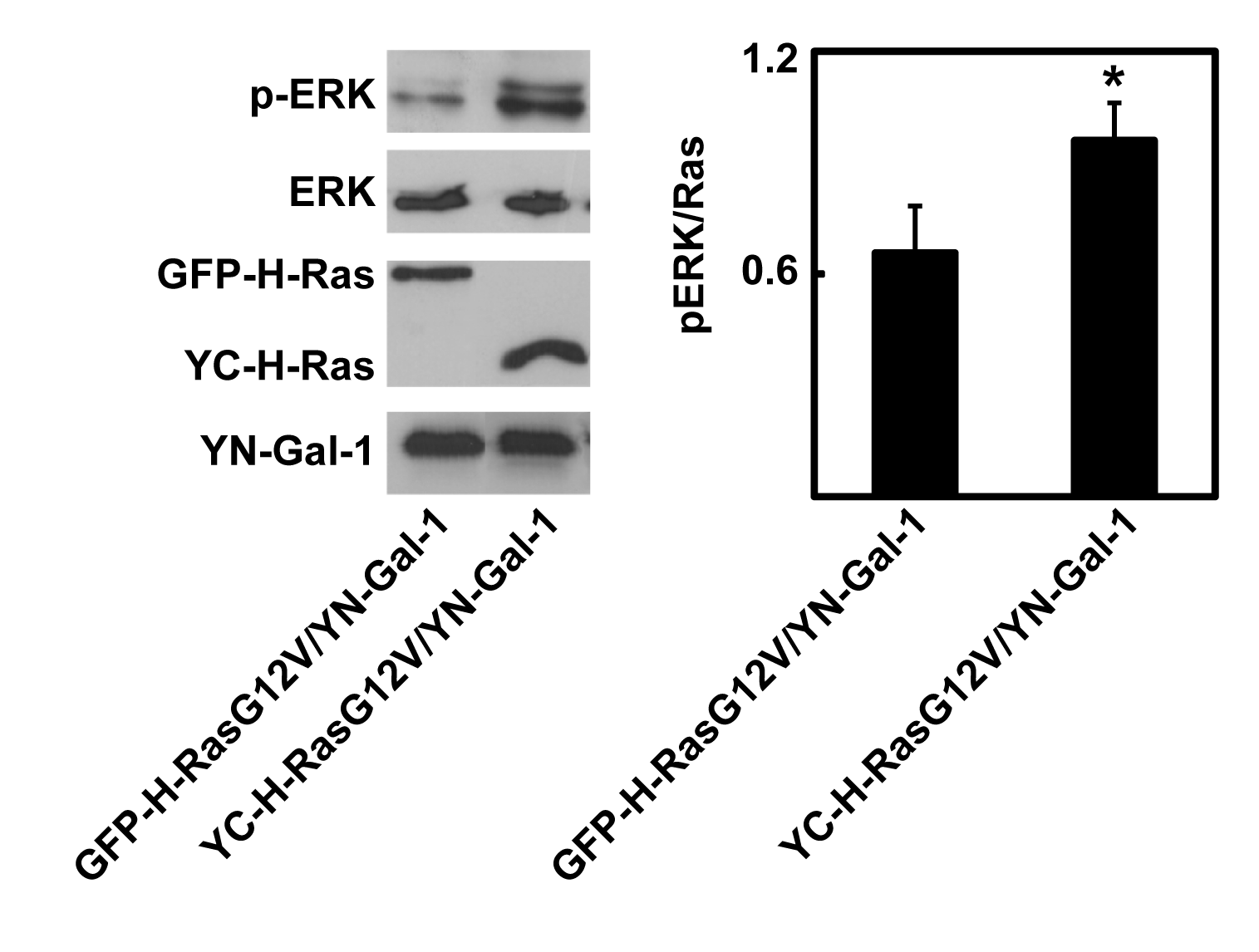

Supplement: Figure S2 — HEK293 cells were co-transfected with YN-Gal1 and GFP-H-RasG12V or YC-H-RasG12V and lysed 48 h later. Aliquots of the cell lysets were subjected to SDS-PAGE followed by immunoblotting with anti-phospho-ERK, anti-ERK, anti-pan-Ras and anti-Gal1 antibodies. Typical immunoblots performed using HEK293 cell are shown (rite) and quantitative densitometry of the levels of phosphor-ERK vs. Ras levels (arbitrary units, means ± SEM, n = 4) are shown (left). P<0.05. (0.20 MB TIF) [file pone.0011991.s002.tif]
